# Survival in general surgery: The female surgeons’ perspective

**DOI:** 10.12669/pjms.39.4.7528

**Published:** 2023

**Authors:** Syeda Saima Qamar Naqvi, Humera Adeeb, Ahsan Sethi

**Affiliations:** 1 Syeda Saima Qamar Naqvi, MBBS, FCPS-General Surgery, MHPE Associate Professor, Baqai Medical University, Karachi, Pakistan.; 2 Humera Adeeb, MBBS, MPhil Community Medicine, MHPE Research Fellow, Khyber Medical College, Peshawar, Pakistan; 3 Ahsan Sethi, BDS, MPH MMED, FHEA, MAcadMEd, FDTFEd, FAIMER Fellow, PhD Associate Professor and Program Coordinator, Health Professions Education, QU Health, Qatar University, Doha, Qatar

**Keywords:** Diversity, Female Surgeons, Gender, General Surgery, Specialty-choices

## Abstract

**Objective:**

Specialty choices in health profession has long been influenced by gender. The field of General Surgery remains the least preferred specialty by females, especially in Pakistan. The objective of this study was to identify the factors leading to success and retention of females in General Surgery in Pakistan.

**Methods:**

Qualitative case study was conducted from February to May 2020 at Khyber Medical University, Peshawar. Ten semi-structured interviews were conducted with purposive sample of female surgeons at various academic position in different tertiary care hospitals of Pakistan. Data were thematically analyzed.

**Results:**

Participants were driven by their passion for surgery, which led them to choose this specialty in the beginning. Their survival in male dominant workplace was made possible by their own personality traits and conducive environment provided by the supervisors and peers. However, a structured mentorship program for females was found lacking.

**Conclusion:**

The necessary ingredients for success are passion and personality traits in any field but attention to nurturing and supportive environment for females in the General Surgery is paramount in determining success. Due consideration to the factors identified in the current study will enhance the retention and success of females in General Surgery.

## INTRODUCTION

Traditionally, females are considered weaker than males, both physically and emotionally.^1^ This has implications with regards to the expectations of society from either gender and for assigning roles and responsibilities to both men and women. In health professions, there are more females in nursing profession, likewise, there are more males in physical therapy. This is also mirrored in the specialty choices made in medicine, where the female doctors mostly prefer to join Gynecology, while male doctors prefer surgery.^2^ Females make up to 70% of the total healthcare workforce worldwide. Out of this, majority of females are restricted to “women’s fields” like gynaecology, obstetrics and nursing. As per Pakistan Medical and Dental Council data for 2016-2018, 58% females were registered as medical graduates. For the same years, 1331 registration certificates were issued for surgical specialties with only 17% females in these specialties.^3^ In a study from Pakistan, only 11.3% female medical graduates preferred General Surgery as career.^4^ Such disparity in gender distribution is not only in General Surgery but across all surgical specialties. Even the number of female surgeons moving from assistant professors (63.1%) to professors (17.8%) is lower than males, reflecting disparity in their carrier progression as well. This disparity is more pronounced in provinces like Khyber Pakhtunkhwa and Balochistan with only 24(6.5%) and 7(3.1%) practicing female surgeons respectively thus suggesting an impact of culture as well.^5^

According to Human Resources for Health Observer Series No. 24. the main reasons of inequalities are gender biased working environments, discrimination and harassment including sexual harassment, gender pay gap and gender disparity in leadership.^6^ Some factors restricting the surgical specialties as career for females include societal expectations of gender role, discouraging attitude about surgery as career for females and responsibility of raising a family.^7^-^9^ These factors extend beyond training and into workplace policies and practices, with limited support system for career development and progression in surgical specialties.

The significant gender inequality in all medical fields and General Surgery in particular has been reported by many studies.^10^,^11^ In predominant Muslim countries, this imbalance has paramount impact, considering the female population preferences for the female healthcare provider on account of religious and cultural factors.^12^ With less female surgeons in the field, these factors increase the likelihood of female patients presenting at an advanced stage of surgical diseases because of reluctance to visit male surgeons until it is inevitable.

International literature has described some factors but on account of socio-cultural differences the validity of these findings may differ. The paucity of local data available on this issue reflects the gap in literature and signifies the need to identify the factors that helped successful female surgeons survive the surgical field in Pakistan. The objective of this study was to identify the factors influencing the retention and success of females in the field of General Surgery.

## METHODS

A qualitative case study with semi-structured interviews was conducted from February to May 2020. Using purposive maximum variation sampling, ten female General Surgeons working at various academic positions at tertiary care hospitals of different cities of Pakistan were interviewed. These cities represented variations of cultural norms and work practices across the country. The participants of the study were all practicing, consultant General Surgeons at academic positions, who completed their postgraduate training from Pakistan. The sample size was not predetermined, instead the data collection and analysis were conducted in an iterative manner until understanding of the phenomenon was achieved, and further interviews elicited no new themes i.e., data saturation. This iterative approach also informed selection of the participants to ensure maximum variation and diversity. All those female surgeons who switched to other medical fields after specialization or currently unemployed, were excluded from the study.

Semi-structured interviews were conducted online after informed written consent. After a thorough literature search, a semi-structured interview guide was developed. Questions were framed around the experiences and perceptions of the female surgeons about challenges due to gender differences during training and their coping strategies. The interview questions were validated through experts. All interviews were conducted in English language. During the interview, probing questions were also added to get a deeper understanding about certain events.

The audio records of interviews were transcribed intelligent verbatim to extract the meaning of spoken words. Thematic analysis was conducted to facilitate identification of themes from the data. The primary codes generated from transcripts were 45 leading to 17 secondary codes after convergence. Finally, five subthemes were developed, which synthesized into three themes.

### Ethical Approval:

The study was approved by the Institute of Health Professions Education and Research. Khyber Medical University, Peshawar, (Ref: 3-9/IHPER/MHPE/KMU/20-18).

## RESULTS

The participants represented almost all the major ethnicities of the country’s population. The working experience of participants in specialty ranged from seven to twenty-five years. The demographic characteristics of study participants are given in Table-I

**Table-I:** Demographic characteristics

Characteristics	Frequency	Percentage (%)
***Age (years)***		
30-40 years	3	30%
40-50 years	6	60%
More than 50 years	1	10%
***Ethnicity***		
Sindh	2	20%
Punjab	5	50%
Khyber Pakhtunkhwa	3	30%
***Work Experience (years)***		
Range (years)	7 - 25	-
Less than 10 years	5	50%
10-20 years	4	40%
More than 20 years	1	10%

We identified five subthemes under three themes as shown in Table-II. The unit of analysis was theme. The author’s background and experience in the specialty might have been reflected in the analysis of transcripts and results.

**Table-II:** Factors affecting success and retention among female surgeons.

Theme	Subthemes	Representative Quotes
Passion drives success	Self-driven	P2: I would have taken guidance if I had to see whether it was feasible for me or not. I always knew that I will be able to do it. P8: I never asked anyone. I had made up my mind that whether anyone likes it or not, I will pursue surgery.
	Skill oriented	P7 : When we started going to wards in 3^rd^ year, I liked to put stitches. In trauma, I was never upset to see blood. When I was working, when one works with adrenaline surge that’s what I used to like. P6 : I just loved watching surgery. Somebody operating or going to the surgical ward, getting the feel of a chest tube or suturing.
Personality Traits	Dominant personality	P1 : It was actually them, who were harassed by me! P10 : I am a little high-rated. I don’t tolerate anyone’s nonsense.
	Imperturbable	P6: (Testimonial written for P6 by a Canadian Surgeon): He wrote a very nice thing. He said, in that kind of an environment… you know… she keeps her head high, without being bothered by them, in an environment where people … you know… try ways to ridicule her or show that she is not good enough or competent enough.
Mentoring	Supportive environment	P2 :Whenever there were small problems, there were many cooperative people to solve those problems. P4 :We were quite close to our supervisor ……Meaning the (working) environment was such that everyone had to report to him, our supervisor. as well.

1. Passion drives success.
2. Personality traits are associated with survival in general surgery.
3. Significance of mentorship in surgical training.

### Passion drives success:

The dominant factor described by all participants was their passion for the specialty. This passion determined their specialty choice very early in their career. This passion was triggered and enhanced by experience of dissection early in medical school. Two of the participants developed interest after exposure to the specialty during clinical rotations. One participant decided to choose surgery after reflecting on the tangible outcomes of surgery versus medicine during apprenticeship. This was described in the participant’s words as shown in Table-II.

### Personality traits associated with specialty choices:

Seven out of ten participants believed that they survived surgery because of their assertive nature. Three participants attributed the differential treatment as they would not fight for surgeries or stand up against any gender discrimination but stayed strong and persistent. Persistence and resilience finally helped participants to overcome such problems. This is reflected by the participant’s words as shown in Table-II.

### Significance of mentorship in surgical training:

All the participants believed they were supported by their peers as well as supervisors at different levels and stages of training which made their success possible. None of the participants had any mentor explicitly assigned to them. Their supervisors were available to support them but none of the participants or the supervisors were bound or exposed to any mentorship program. This is shown in the words of participants in Table-II.

## DISCUSSION

All participants attributed their success in the General Surgery speciality to passion and personality traits. All of them had a strong desire to become a surgeon and their passion for this profession made it easy for them to overcome the hurdles that came along. An assertive and resilient personality were the helpful attributes to cope with stressful working environment. This, when reinforced with a conducive working environment provided by their colleagues as well as supervisors, led to their success in general surgery. Although none of the participants was assigned any mentor at any stage of their training, all of them agreed on having good support from their supervisors whenever they were approached.

Literature has described that passion for surgery is one of the most common driving forces for choosing this field.^13^ A study conducted on 18 Canadian medical schools revealed that out of 2168 respondents only 21% chose surgery as their future career choice. For all of them the main driving force was their personal interest in this field.^14^ The “want” to become a surgeon drives both males and females equally to pursue this career. For females this drive becomes more important as they have to adjust to a male dominant environment.^15^ If “the heart is on fire” nothing can stop it.^16^Another driving and encouraging factor is demand for female surgeons by patients, preferably when operating procedure is female gender specific.^17^ The demand and societal expectations is another reason for choosing surgery as career.^18^,^19^ By highlighting the positive aspects of career in surgery, passion and love for surgery can be developed in medical graduates.

Passion coupled with personality traits can have a strong impact on females’ success in General Surgery. Lauren Crawford, a female plastic surgeon, in her article “10 secrets to success as a female surgeons” says that female should grow a “thick skin” and a “thicker spine” to survive in surgery.^20^ In a recent study, conducted on about 600 male surgeons, it was identified that female surgeons were more likely to have “openness” and “extroversion” as a personality trait as compared to population average.^21^ The psychological evaluation for personality traits may help in future specialty choices. This may help in lowering gender’s chance of attrition.

The importance of mentoring program in General Surgery cannot be emphasized enough.^22^ The disparity in mentoring relationships and bias in provision of opportunities during residency leads to lower job satisfaction among female surgeons.^5^,^9^ A study conducted in Switzerland, reported a positive impact on academic career of female surgeons, who had a mentor-mentee relationship.^23^ This same study, however, also indicated that only half of the participants had access to a mentor. Another study conducted in United Kingdom also gives similar findings, where out of 565 trainees only 48.7% reported to have a mentor.^24^ A study by Karam A et al, reported availability of less mentorship to females (45%) as compared to male residents/surgeons (51%).^25^ There is a need for formal institutional support groups and structures that can help reduce stress, ensure work-life balance and prevent burnout among female surgeons.^23^ Moreover, a structured mentoring program for the female surgical residents and also training the supervisors, on how to be effective mentors, can further enhance the retention and success of females in General Surgery.^23^,^26^

The participants of this study belong to different ethnic and cultural strata of the society. The participants experienced exposure to different cultural norms during training and thus are able to share the variety of work-place based issues. The participants are successful practicing surgeons; hence the opinions are less likely to be influenced by unnecessary blame on minor deterrents seen in any work-place with gender inequalities.

### Limitations:

The main limitation of this study is non-availability of participants from Balochistan. However, this region has some similarities with neighboring Sindhi and Pathan cultures that is well represented.

## CONCLUSION

Passion, Personality traits and effective mentoring are essential for Female Surgeons. They need a conducive environment for survival in General Surgery. Having mentors may help females make right specialty choices earlier in the career. Due consideration to the factors identified in the current study will help enhance promotion of equitable system for the retention and success of females in General Surgery.

### Authors Contributions:

**SSQN** and **AS**: Conceptualized the study.

**SSQN**, **AS** and **HA**: Designed the study.

**SSQN**: Conducted the interviews.

**SSQN** and **HA**: Prepared the first draft.

**AS** reviewed and revised critically for important intellectual content.

All the authors approved the final version and agree to be accountable for all aspects of the work.

All the authors were involved in data analysis and interpretation.
